# Editorial: The Balanced Triad of Perception, Action, and Cognition

**DOI:** 10.3389/fpsyg.2016.00991

**Published:** 2016-07-08

**Authors:** Snehlata Jaswal

**Affiliations:** Psychology, Cognitive Science, Indian Institute of Technology JodhpurJodhpur, India

**Keywords:** perception, action, cognition, embodied cognition, imitation, self-concept

Conceptual distinctions between perception, action, and cognition are convoluted in real world proceedings, as we meander through life, tied to past thoughts and actions, oriented toward future goals, guided by current perceptions. The research topic “Perception, Action, and Cognition” aimed to go beyond the already established perception-action links to study the role of cognitive mechanisms in this triad.

A popular way of conceptualizing the triad of perception, action, and cognition is the idea of embodied cognition. The first section entitled “Embodied cognition” presents seven articles related to this concept. In the leading article, Hommel develops an efficient way to conceptualize embodied cognition using the Theory of Event Coding (TEC) framework. Arguing against the anti-cognitivist stance of many embodiment theorists, he firmly puts cognition back in the perception-action link, by maintaining the importance and involvement of internal representations in the production of actions. Vernon et al. propose that perception-action coupling is not only manifest in the behavioral arena, but also shows up in the internal processes of the agents, particularly those related to the self. The self-organizing, self-producing, and self-maintaining processes explain the reciprocity of perception and action. The sense of presence, which is the focus of the article by Triberti and Riva actually implies the presence of the “self” in the environment where perception-action coupling is manifest. Brizio and Tirassa go a step further to expound on how the mind is rooted in the self, particularly the biological self. They offer a taxonomy of control systems based on whether they are intentional, non-intentional, or meta-intentional, drawing on arguments from diverse disciplines ranging from psychology to biology to philosophy to artificial intelligence. The next two articles provide evidence for embodied cognition from clinical samples. Dreyer et al. report evidence from two patients with focal lesions, regarding sensorimotor systems in the cortex being crucial for the processing of semantic concepts, suggesting that without intact perceptual-motor systems, meaningful cognition is all but impossible. Wolpe et al. show that Parkinsons' Disease (PD) patients with higher levodopa dose equivalent show an abnormally high awareness of their actions and their positive outcomes, providing evidence for intact action systems feeding perceptual awareness. Finally, evidence regarding action being important for cognition also comes from a cross cultural study by Wang et al. who studied children's use of imitation in learning how to categorize objects. They propose that imitation of an action leads to direct experience, which in turn stimulates rule learning/categorization. This process is the same across the two cultures studied—Chinese and US.

The rest of the articles are ingenious forays into the socio-cultural realm. The second section of the research topic is, therefore, titled “The external world and inner reality.” We begin the section with mirror mechanisms in the brain, the biological underpinnings of social perception and interactions. Volta et al. report an fMRI study that showed similar brain areas to be activated whether participants were actually walking or merely observing someone else walking, thus supporting the idea of mirror mechanisms for walking. However, differences in brain areas activated were evident between two conditions—one in which a video of open countryside was played and the other in which the closed space of a corridor was depicted, indicating the strong influence of external context on brain processing. Desanghere and Marotta show that the physical shape of an object and its center of mass, are distinct influences, in how we look at it, and grasp it. This study independently manipulated physical shape and center of mass of an object, and thus used irregularly shaped objects to study the effect of these physical features on gaze and grasp. This study is a good illustration of how the same percept may lead to different reactions in different parts of the body perhaps because the relevance of the percept to the body part is different. In another study demonstrating the effect of perceptual cues on Simon task performance, Umemura demonstrates that stimulus locations in 2D and in 3D space give independent cues for task performance to the participants. This seems understandable; for pictorial cues are sufficient for 2D locations, but 3D locations can be precisely pinpointed only by the convergence of the eyes. Studying an important variable in our social interactions, Kuraguchi and Ashida focus on detection of beauty and cuteness. They found that whilst beauty can be equally discerned in central as well as peripheral vision, the detection of cuteness declines in peripheral vision as compared to central vision, especially in males. In another study of face processing, it becomes clear why autism is associated with difficulties in the socio-emotional sphere. Ujiie et al. found that university students scoring high on Autism Spectrum Quotient, a measure of autistic traits, show abnormal audio-visual speech integration, probably because they do not show adequate processing of global facial configurations. All these studies show how changes in the external environment result in behavioral/ motor changes.

In turn, the influence of actions or their outcomes on socio-cognitive processing is clear from the next two studies. Sun et al. compared decision times on a simple task presented on the winners' or the losers' side of the screen. Results showed an attention bias in the onlookers for the losers' side of the screen. Could this be the basis of empathy/sympathy in social situations? Using a Rapid Serial Visual Presentation paradigm, Kihara et al. demonstrate that when participants were asked to change a digit stream to a letter stream by pressing a button and identify four successive targets, successful target identification was linked to pupil dilation (a measure of the involvement of the locus coeruleus-noradrenaline system at the neural level). Thus, voluntary action initiated this neural substrate of transient visual attention.

The “self” emerges as a strong player in this section as well. Spape et al. present a new paradigm utilizing “avatars” or representations of the self in virtual reality, which can be used by researchers in cognition, social psychology, and human computer interaction. The study also augments our conceptualization of executive control, by showing how changes in the gender of the “avatars” can disrupt conflict adaptation in the virtual reality version of the Simon task. Zhang et al. use the virtual hand illusion to demonstrate that the bodily self is not a definite exact entity, rather, body representations are dynamic and are constantly updated by current inputs from the context. Imbir demonstrates the effect of subjective significance on the rational mind, and arousal on the experiential mind, using the emotional stroop task. The control of “reason,” therefore, is dependent on whether we perceive a situation to be of significant or not. Finally, the review by Kavanagh and Winkielman suggests mimicry as an implicit but adaptive mechanism that underlies affiliation between the model and the mimic, and also signals affiliation with the group.

All these articles are heartening examples of work that goes much beyond establishing the perception-action link. Most contributions specify the mechanisms for this link, or the antecedent or consequent conditions of the link. For example, the articles in the first section propose the self as the unifying entity for perception and action. In the second, whereas some articles study the effect of perceptual factors on behavior, others specify how action influences attention/perception, and still others focus on the mediator. i.e., person involved. A perusal of all articles in this research topic reveals that perception, action, and cognition, are an unbreakable triad. Many manifest links have been established, and many other latent ones need to be explored in future research.

## Author contributions

The author confirms being the sole contributor of this work and approved it for publication.

### Conflict of interest statement

The author declares that the research was conducted in the absence of any commercial or financial relationships that could be construed as a potential conflict of interest.

